# Low concentration of hyaluronidase for oocyte denudation can improve fertilization rates and embryo quality

**DOI:** 10.5935/1518-0557.20170008

**Published:** 2017

**Authors:** Bernardo R L de Moura, Mariana C A Gurgel, Suelen PP Machado, Priscila A Marques, Juliana R Rolim, Marcelo C de Lima, Lister L Salgueiro

**Affiliations:** 1 Clínica Fértilis de Medicina Reprodutiva, Sorocaba, SP, Brazil; 2 Universidade Anhembi Morumbi, São Paulo, SP, Brazil

**Keywords:** ICSI, Hyaluronidase, oocyte denudation, *cumulus*- oocyte complex

## Abstract

**Objective:**

Hyaluronidase enzyme is an extremely important factor for the process of oocyte denudation, but little is known about its negative effects.

**Methods:**

This prospective randomized study analyzed the results of using different concentrations of hyaluronidase (Diluted: 8IU/mL and Normal: 80IU/mL) used for denudation of sibling-oocytes for 22 women undergoing treatment for assisted reproduction by ICSI. A total of 192 oocytes were injected, being 104 for group I (diluted) and 88 for group II (normal). We analyzed fertilization rate, cleavage, embryo quality at 48 and 72 hours and number of transferred embryos in each group.

**Results:**

The diluted enzyme group showed better results in fertilization rates (92.3% vs. 80.6%), mean cleavage (4.18 ± 2.57 vs. 3.09 ± 1.90), in 48-hour embryos A and A + B (60.9% vs. 44.1% and 90.2% vs. 82.3%) and at 72 hours (45.6% vs. 36.8% and 77.1% vs 66.2%), and number of embryos selected for transfer (61.8% vs. 38.1%). The overall pregnancy rate was 59.1%.

**Conclusion:**

This study demonstrates that the use of 8 IU/mL of hyaluronidase, according to the following protocol, is beneficial and can be successfully used for oocyte denudation, and it is also economically advantageous to the laboratory

## INTRODUCTION

With the advent of Intracytoplasmic Sperm Injection technique (ICSI) there was a great advance in assisted reproductive technology (ART), and thousands of infertile couples were benefited. The development of this technique led to the need for a better classification of oocytes, as well as the choice of the most capable sperm for fertilization purposes.

The success of this technique depends of some factors, such as specific equipment, competent embryologists (Gordts *et al*., 1995), oocyte quality (Liu *et al*., 1995), zona pellucida penetration, oolema rupture (Nagy *et al*., 1995; Palermo *et al*., 1996) and sperm viability (Nagy *et al*., 1995).

Not all oocytes obtained through follicular aspiration have competence and maturity to be fertilized; therefore, the oocytes must be previously classified to the moment of injection according to its maturity degree. However, this classification is only possible after the removal of the cumulus and corona cells surrounding the oocyte, and this process is called oocyte denudation (De Vos *et al*., 2008).

Oocyte denudation is a crucial process that demands extreme caution and experience from the embryologist, because it combines two actions: one mechanic and one enzymatic. Therefore, there are some criteria needed to preserve cell quality. After denudation, the ideal is to see the extrusion of the first polar body, which means that the oocyte is in the second metaphase. This oocyte cleaning process (denudation) is extremely important, because besides classifying the oocyte, it allows the visualization of morphological alterations, such as intracytoplasmic granulation, fragmentation of the polar body and increased perivitelline space, then enabling the selection of more viable oocytes for fertilization (Ebner *et al*., 2006).

The enzyme used for denudation is hyaluronidase, this enzyme digests the hyaluronic acid interspaced between the cumulus cells (Mahadevan & Trounson, 1985). In many laboratories, the standard concentration used is 80 IU/mL, as suggested by Joris *et al*. (1998). Previously to the establishment of this concentration, the increase in degeneration and parthenogenetic problem rates were frequent with the use of higher concentrations of this enzyme. The results published by Van de Velde *et al*. (1997) found that lower concentration of hyaluronidase, using a higher caliber pipette, can effectively decrease the time of oocyte exposure to the enzyme. Therefore, using a protocol with short exposure for removal of cumulus cells is fundamental, because although hyaluronidase dissociates the cells from the cumulus artificially, it can also damage the oocyte in several areas.

On the other hand, some authors believe that the long exposure (around 30 min) together with high concentrations of hyaluronidase (50-250 IU/mL) is not a triggering factor for the parthenogenetic activation of the oocyte. However, there is a lack of understanding about the effects of the enzyme on human oocytes (Laufer *et al*., 1984; Mahadevan & Trounson, 1985; Pickering *et al*., 1988; Abramczuk & Lopata, 1990).

Until now, few studies have tested the possible effects of different concentrations of hyaluronidase in sibling-oocytes. This study aimed to evaluate the effectiveness of the enzyme and compare different laboratorial parameters such as fertilization and cleavage rates, embryo quality and number of transferred embryos using two different concentrations of hyaluronidase (80 and 8 IU/mL) in sibling-oocytes.

## MATERIALS AND METHODS

The protocols and procedures used in the present study were approved by the Institutional Board Review (IBR) from Fértilis Clinic of Assisted Reproduction. This prospective and randomized study included twenty-two patients submitted to Assisted Reproduction treatment between January and May 2011.

A randomized sibling-oocyte trial was started to evaluate the effectiveness and possible influence of diluted concentration of 8IU/mL of hyaluronidase when compared with the standardized concentration of 80IU/mL of hyaluronidase. Patients with over four fresh oocytes were included at this study, regardless other associated clinical factors. As for exclusion criteria, we list: patients with less than four oocytes, total failure of fertilization after ICSI and absence of embryos for transfer. By using a pair of dishes for sibling oocytes from each patient in a paired study design, differences in patient characteristics, such as female age, rank of trial, duration and origin of infertility, were taken off.

All the patients were submitted to controlled ovarian hyperstimulation, using long term protocol with Gonadotropin Releasing Hormone (GnRH) agonist, as suggested by previous studies (Dozortsev *et al*., 2004). Oocytes were recovered by follicular aspiration guided by transvaginal ultrasound, 35 hours after the administration of Human Chorionic Gonadotropin (hCG).

After aspiration, follicular liquid checking and separation of oocytes, the denudated hyaluronidase (Hyase, Irvine Scientific, California, USA) diluted in modified HTF medium (mHTF) (HEPES-buffered; Irvine Scientific, California, USA) supplemented with 15% SSS (Synthetic Serum Substitute, Irvine Scientific, California, USA), at a final concentration of 8 IU/mL. In group II (GII), oocyte denudation was performed with 80 IU/mL hyaluronidase. In the case of having an odd number of oocytes, the supernumerary was placed on GI, according to a standardized random group decision. Aiming to maintain impartiality in the separation of oocytes, the focus of the stereomicroscope was withdrawn so there was no clear visualization of the cumulus - oocyte complex (COCs).

The denudation process was accomplished in two steps. The first (enzymatic) was performed subsequently to the separation of the groups. Separately, COCs from GI and GII were immersed in hyaluronidase in the correspondent concentration for each group, with a large diameter glass Pasteur pipette (5.75" IVF Pasteur Pipets, Origio, Denmark) performing movements of entry and exit from the pipette for 30 seconds, followed by washing in mHTF medium supplemented with 15% SSS to remove the excess enzyme. Culture was performed in the groups GI and GII in a double gap dish (BD Falcon 35-3037, Nova Jersey, USA) with 1mL of SSM (Single Step Medium, Irvine Scientific, California, USA) supplemented with 15% of SSS and covered with mineral oil (Oil for embryo culture, Irvine Scientific, California, USA).

The oocytes remained in culture incubators (Forma Series II model: 3110; Water-Jacketed CO_2_ Incubators, USA) at an atmosphere of 5.5% CO_2_, at37ºC for one hour and then submitted to the mechanic step of denudation. On the stereomicroscope, we found COCs on the bottom of the plate, with few cells left around the oocyte in both groups. Remaining cells were removed using a manipulation pipette (Flexipet Adjustable Handle Set, Cook Medical, Indiana, USA) with the use of a 130 µm internal diameter tip (Stripper Tips - Handle Cook, Indiana, USA). Oocytes were moved in and out of the pipette until total denudation and visualization for classification of nuclear maturation. By the end of denudation and classification, the oocytes were placed in 50 µL drops of SSM with 15% of SSS covered with 4 mL of mineral oil and incubated until ICSI.

ICSI was performed in mature oocytes three hours after the aspiration as previously described by Palermo *et al*. (1992) and Abdelmassih *et al*. (1996). Fertilization and cleavage checking were always performed by the same embryologist, without identifying the groups. The criteria used were the same as described by Dozortzev *et al*. (2004).

For embryo transfer (ET), a catheter (Sidney IVF - Handle Cook, Indiana, USA) connected to a Terumo, 1.0 mL syringe (Terumo Medical do Brasil, São Paulo, Brazil) was used. The catheter was previously washed with transfer medium (mHTF + 15% SSS) than the embryos were loaded and injected into the uterus under pelvic ultrasound guidance. Vaginal administration of progesterone was initiated upon the oocytes aspiration day (600 mg/day Utrogestan; Besins Laboratories, Paris, France) and was maintained until pregnancy evaluation (12 days after the transfer). Serum levels of β-hCG were measured on day twelve after the transfer. Clinical pregnancy was determined by visualization of heart beat trough ultrasound within six weeks' gestation.

Statistical significance was determined by Wilcoxon test. Differences were considered significant for *p* ≤ 0.05.

## RESULTS

A total of two hundred twenty-one oocytes were aspirated from twenty-two cycles of IVF-ICSI analyzed. One hundred ninety-two oocytes were classified as MII and injected, being 104 (54%) in GI and 88 (46%) in GII. Data related to average age, oocytes, transferred embryos and pregnancy rates are described on Table 1.

**Table 1:** General characteristics of the participants' cycles in the study

Variable	N
Total *#* of Patients	22
Age^a^	33.7 ± 5.65
Total oocytes	221
Oocytes^a^	10.04 ± 4.50
Total MII oocytes^c^	192
Injected (MII)^a,c^	8.72 ± 4.34
Total Embryos Transferred	55
Embryos Transferred^a^	2.5 ± 0.67
Pregnancy rates (%)^b^	59.1 (13/22)

a Values are Mean ± Standard Deviation (SD)

b Mean percentage per cycle

c MII, mature metaphase-II oocytes

Data on laboratorial parameters analyzed in both groups are on Table 2. As demonstrated, the first group showed better results when compared with the second group for the following parameters: fertilization 92.3% (96/104) vs. 80.6% (71/88), *p* = 0.006), grade A embryos (with best morphology) in 48 hours (60.9% (56/92) vs. 44.1% (30/68), *p* = 0.006) and 72-hour cleavage (45.6% (42/92) vs. 36.8% (25/68), *p* = 0.044) besides higher index of embryos selected for transfer in GI (61.8% (34/55)) when compared with GII (38.8% (21/55)). For better analysis of embryo quality, data of embryos with the best (grade A + B) and worst (grade C + D) quality were joined for both analysis at 48 and 72 hours, and, again GI showed better results (48hs = 3.77 ± 2.39 vs. 2.55 ± 1.63, *p =* 0.004 and in 72hs = 3.23 ± 2.37 vs. 2.05 ± 1.79, *p* = 0.006).

**Table 2:** Average value of the results per group

Variable	GI (Diluted)	GII (Control)	*p*
Average rate of injected oocytes	4.72 ± 2.35	4.0 ± 2.09	0.004
Fertilization average rate	4.36 ± 2.49	3.22 ± 1.87	0.006
Cleavage average	4.18 ± 2.57	3.09 ± 1.90	0.010
48 hours' evaluation rate			
GRADE A	2.54 ± 2.21	1.36 ± 1.67	0.006
GRADE B	1.23 ± 1.44	1.18 ± 1.05	NS
GRADE C	0.36 ± 0.58	0.41 ± 0.91	NS
GRADE D	0.04 ± 0.21	0.14 ± 0.35	NS
GRADE A + B	3.77 ± 2.39	2.55 ± 1.63	0.004
GRADE C + D	0.41 ± 0.67	0.55 ± 1.14	NS
72 hours' evaluation rate			
GRADE A	1.91 ± 1.87	1.13 ± 1.35	0.044
GRADE B	1.32 ± 1.21	0.90 ± 1.01	NS
GRADE C	0.73 ± 0.83	0.73 ± 0.98	NS
GRADE A + B	3.23 ± 2.37	2.05 ± 1.79	0.006
GRADE C + D	0.96 ± 1.17	1.05 ± 1.29	NS
Average nº of transferred embryos	1.59 ± 0.67	0.95 ± 0.35	0.005

Each value represents mean ± standard deviation, NS = not significant

Each value represents mean ± standard deviation, NS = Not Significant

There was no statistic difference between the groups regarding embryo quality parameters in grades B, C, D and C + D upon evaluations at 48 and 72 hours. An average of 2.5 ± 0.67 embryos were transferred, being the maximum of 4 transferred embryos, varying according to patient's age and characteristics. The general pregnancy rate of the patients in the study was 59.1% (13/22).

## DISCUSSION

Our objective was to test a lower concentration of enzymes and its effectiveness at denuding, COC and compare different laboratorial parameters such as fertilization and cleavage rates, embryo quality and number of transferred embryos. The results of the present study demonstrate that the diluted enzyme is effective for denuding oocytes prior to ICSI treatment in this protocol. Furthermore, the data suggests that this lower concentration of enzyme significantly improves fertilization rates and positively affect embryo development compared to the standard concentration product: Hyase.

The average rate of injected oocytes was significantly different between the groups because of an odd number of oocytes, and it was randomly decided that every unpaired oocyte would be designated to the first group. Although, it was not possible to assess pregnancy rate in each group, to maintain the chances of pregnancy for the patients, always the best morphology graded embryos were selected for transfer, independent of the group where they were.

The times exposed to each solution were always the same, thirty seconds, between the two groups, indicating that lower concentration is as effective as the traditionally used hyaluronidase concentration for disrupting cumulus cells. However, the time oocytes are in each solution is subjective in that each embryologist uses different criteria before oocytes are removed. Many clinics use a hyaluronidase concentration of 80 IU/mL, which would lead to shorter exposure times to hyaluronidase. Van de Velde. (1997) found that a lower concentration of hyaluronidase and a pipette of larger diameter could effectively limit the exposure time of oocytes to hyaluronidase.

Furthermore, those authors discuss the fact that using lower concentrations of hyaluronidase could considerably reduce parthenogenic activation rates. In this study, we could observe that in the group exposed to a lower concentration of hyaluronidase had better fertilization rates and higher embryo quality. This leads us to believe that the enzyme is not only related to parthenogen but also to toxicity, which could lead the oocyte to lose its quality, fertilization and cleavage viability, besides embryo quality, so finding a protocol that allows for shorter exposure and effective removal of COC is critical (Van de Velde *et al*., 1997).

Joris *et al*. (1998) suggested 80 IU/mL as an ideal concentration. Ever since only few studies were performed aiming to correlate the possible toxic effects of hyaluronidase in several laboratorial parameters of *in vitro* fertilization. Other studies reported some trials using lower concentrations, such as 40 and 20 IU/mL, but they only compared this bovine-derived enzyme to the recombinant enzyme, not supporting our findings, but they also suggest some possible effects of toxicity that could influence fertilization rates and embryo quality (De Vos *et al*., 2008; Taylor *et al*., 2006).

Hyaluronidase is, without a doubt, extremely important, because it aids in oocyte activation and it improves fertilization rates (Tesarik *et al*., 1994; 1995). However, there is little literature data about its possible negative effects and its ideal concentration for oocyte denudation. According to Palermo *et al*. (1996), about 17% of oocytes present parthenogenetic activation during denudation, which leads to a constant search for improvements in this process.

The results of the present study suggest that the lower concentration of enzyme, at 8,0 IU/mL (Hyase), is effective for use in an ICSI treatment program for obtaining high fertilization and developmental rates, together with better embryo quality. This study also shows an economic alternative, since the same amount of hyaluronidase used nowadays for denudation in one cycle, could be potentially used for ten cycles. However, future studies are needed to support these findings.
